# A predictive score for 30-day survival for patients undergoing major lower limb amputation for peripheral arterial obstructive disease

**DOI:** 10.1007/s13304-021-01085-5

**Published:** 2021-06-13

**Authors:** Marco Franchin, Vincenzo Palermo, Carlo Iannuzzi, Nicola Rivolta, Gaddiel Mozzetta, Matteo Tozzi, Ruth L. Bush, Gabriele Piffaretti

**Affiliations:** 1 Vascular Surgery, Department of Medicine and Surgery, University of Insubria School of Medicine, Via Guicciardini, 9, 21100 Varese, Italy; 2 College of Medicine, University of Houston, Houston, TX USA

**Keywords:** Major amputation, Gangrene, Critical limb ischemia, Acute limb ischemia, 30-day survival

## Abstract

To analyze outcomes following major lower extremity amputations (mLEAs) for peripheral arterial obstructive disease, gangrene, infected non-healing wound and to create a risk prediction scoring system for 30-day mortality. In this single-center, retrospective, observational cohort study. All patients treated with above-the-knee amputation (AKA) or below-the-knee amputation (BKA) between January 1st, 2010 and June 30th, 2018 were identified. The primary outcome of interest was early (≤ 30 days) mortality. Secondary outcomes were postoperative complications and freedom from amputation stump revision/failure. We identified 310 (77.7%) mLEAs performed on 286 patients. There were 188 (65.7%) men and 98 (34.3%) women with a median age of 79 years (IQR, 69–83 years). We performed 257 (82.9%) AKA and 53 (17.1%) BKA. There were 49 (15.8%) early deaths, which did not differ among the age quartiles of this cohort (15.4% vs. 14.3% vs. 15.4% vs. 19.5%, *P* = 0.826). Binary logistic regression analysis identified age > 80 years (OR 2.24, 95% CI 1.17–4.31; *P* = 0.015), chronic obstructive pulmonary disease (OR 2.12, 95% CI 1.11–4.06; *P* = 0.023), and hemodialysis (OR 2.52, 95% CI 1.15–5.52; *P* = 0.021) to be associated with early mortality. The final score (range 0–10) identified two subgroups with different mortality at 30 days: lower-risk (score < 4, 10.8%), and higher-risk (score ≥ 4: 28.7%; OR 3.2, 95% CI 1.63–6.32; *P* < 0.001). In our experience, mLEAs still have a 14% mortality rate over the years. Our lower-risk group (score < 4) is characterized by a lower rate of perioperative death and longer survival.

**Graphic abstract:**

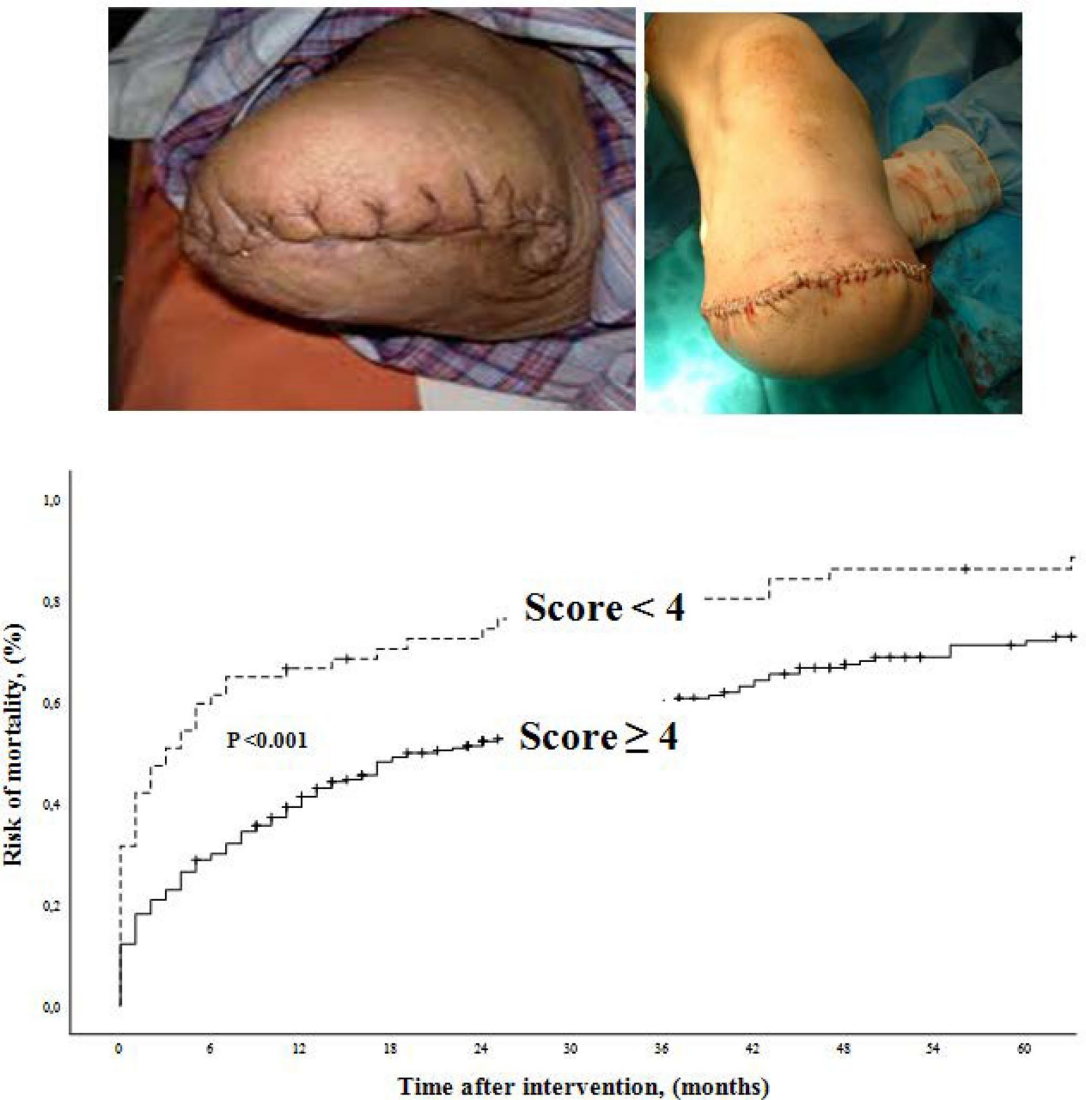

## Introduction

Patients who undergo major lower extremity amputation (mLEA) secondary to peripheral arterial occlusive disease (PAOD) have been reported to have a poor prognosis, likely due to the significant comorbidities and risk factors that exist in this population [1, 2]. Despite the decline in postoperative short-term mortality, no significant uniform improvement over time was observed at mid-to-long term follow-up [3, 4].

Prevention, early diagnosis, and aggressive medical and surgical treatment for patients with severe PAOD or infection has been studied, however, mortality rates remain high [3, 4]. Therefore, perioperative risk stratification may play a key role in patient counseling and improving postoperative outcomes [5–7].

The aims of this study were to analyze major clinical outcomes while identifying predictors of mortality to generate a risk index score in a contemporary cohort of patients after a first amputation for PAOD and/or infection.

## Materials and methods

### Study cohort

**Fig. 1:**
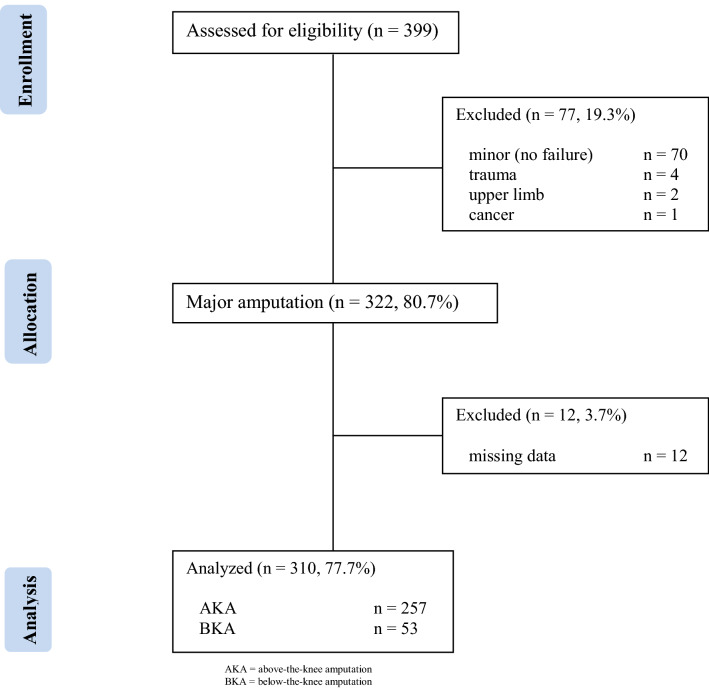
Consort diagram of lower extremity amputations during the period of study (January 1st 2010–June 30th 2018; *n* = number; *AKA* above-the-knee; *BKA* below-the-knee)

This is a single-center, retrospective, observational cohort study from a tertiary referral university hospital. We followed the checklist of items recommended by the STROBE statement [8]. For this study, all patients treated with above-the-knee amputation (AKA) or below-the-knee amputation (BKA) between January 1st, 2010 and June 30th, 2018 were identified. Post-hoc analysis identified those who underwent mLEA for PAOD, gangrene, infected non-healing wound. Medical records were reviewed by two senior surgeons (MF and GP). A consort diagram indicating all patients who underwent amputation during the period of study, including the study cohort from which this series was derived is reported in Fig. 1. People with a previous amputation distal to, and including, ankle disarticulation were included in the final analysis. Data collected included demographics, co-morbidities, severity of PAOD, surgical history, blood test (haemoglobin, leukocyte count, C-reactive protein, albumin) operative details (type of anaesthesia, duration of intervention, level of amputation), as well as postoperative events (amputation revision and mortality) during hospitalization and follow-up period. Owing to the retrospective nature of the present study, local Ethical Committee approval was not necessary according to the Italian National Policy in the matter of Privacy Act on retrospective analysis of anonymized data.

### Indication for interventions

Informed consent for prospective data collection and surgical intervention was signed by each patient. The interventions were performed according to the national guidelines of the Italian Society for Vascular and Endovascular Surgery (SICVE), which are consistent with the clinical practice guidelines on the diagnosis and treatment of PAOD of the European Society for Vascular Surgery (ESVS) [9, 10]. In general, primary amputation was performed in patients with extremely limited life expectancy, extensive necrosis or infectious gangrene, non-ambulatory status. Secondary amputation was performed when revascularization attempts failed and re-intervention was no longer possible because of the absence of a target vessel, or when the limb continued to deteriorate because of infection or necrosis despite adequate blood flow and optimal medical management. The level of amputation was determined based on the clinical judgment of the multidisciplinary team (vascular surgeon and anesthesiologist). Factors assessed included pre-existing limb-threatening ischemia and/or infection and decreased likelihood of salvageability. Computed tomography angiography magnetic resonance, or conventional angiography was not routinely performed to dictate the level of amputation. Indications for the choice of AKA rather than BKA included extensive gangrene or infection, flexion contracture of the knee ≥ 30°, or pre-existing prolonged non-ambulatory status. The type of anesthesia (general vs. spinal/epidural) was at anesthesiologist’s judgment. All patients received perioperative antibiotics. Patients who had no tissue loss or infection received short-term use of cefazolin (2gr b.i.d.; Cefamezin—Pfizer; Milan—IT). Those with tissue loss or infection received broad-spectrum antibiotics consisting of a glycopeptide (Vancotex®—Pharmatex; Milano—IT) and penicillin/beta-lactamase inhibitor (Textazo®—Pharmatex; Milano—IT), unless there was microbiological data already available with drug sensitivities. Postoperatively, electrocardiograms and cardiac enzyme analysis were performed. Generally, routine intensive care unit admission was not indicated per protocol, while rehabilitation transfer was offered to almost all patients to reach personalized outcomes.

### Definition and primary outcomes

Medical comorbidity grading system and operative outcomes were defined according to the Society for Vascular Surgery (SVS) [11]. Chronic kidney disease was defined in agreement with the clinical practice guidelines of the Kidney Disease Improving Global Outcomes [12]. Chronic obstructive pulmonary disease was defined accordingly to the GOLD executive summary [13]. Rutherford classification was used to define critical limb ischemia (CLI) or acute limb ischemia (ALI) [9–11]. Patient’s frailty was assessed using the modified Frailty Index (mFI) [7]. The mFI consists of eleven parameters which generate a frailty score, by giving 1 point for each component and a maximum score of 11.Frailty patient was classified who had a cutoff of mFI > 2 [7]. Failure of the initial amputation was defined as the need for conversion to a higher level. Conversion of BKA to AKA was performed for failed BKA, defined as the presence of non-healing tissues with extensive deep infection or wound disruption, or extensive stump tissue ischemia. The Clavien-Dindo grading system was used to classify postoperative complications [14]. Follow-Up Index (FUI) describes follow-up completeness at a given study end date as a ratio between the investigated and the potential follow-up period [15]. Through December 2020, information on reintervention, vital status, and date of death of individual patients were validated by death certificate, electronic charts managed by the regional health care system, or certified data from Emergency Department admission. For this study, the primary outcome of interest was early (≤ 30 days) mortality. Secondary outcomes were postoperative complications and freedom from amputation stump revision/failure. Time to death was calculated from the date of the first amputation.

### Statistical analysis [16]

Clinical data were recorded and tabulated in Microsoft Excel (Microsoft Corp—Redmond; Wash—USA) database. Statistical analysis was performed by means of SPSS 26.0 for Windows (IBM SPSS—Chicago; Ill—USA). Considering the reported median 9% rate mortality at 30-days, an *ɑ* cut-off of 0.05and a power of 90%, for a 15% expected mortality our cohort would have enrolled a total of 288 patients. Categorical variables were presented using frequencies and percentages. Continuous variables were presented with mean ± standard deviation (SD), or median with interquartile range (IQR) and ranges, based on data distribution. Categorical variables were analyzed with the *χ*^2^ test, and Fisher’s exact test when appropriate. Continuous variables were tested for normal distribution by the Shapiro–Wilk’s test and compared between groups with unpaired Student’s *T*-test for normally distributed values; otherwise, the Mann–Whitney *U* test was used. Tukey’s honest significance test was used as single-step multiple comparison to find a significant difference among means. Univariate analysis was used to identify potential predictors of mortality at 30-days. Associations that yielded a *P* value < 0.20 on univariate screen were then included in a binary logistic regression analysis using the Wald’s forward stepwise model. The strength of the association of variables with mortality was estimated by calculating the odd ratio (OR) and 95% confidence intervals [(95% CI): significance criteria 0.25 for entry, 0.05 for removal)]. Model discrimination was evaluated by using the area under the receiver operating characteristic (AUROC) curve, with ≥ 0.7 being considered significantly accurate. A risk score for mortality at 30-days was then constructed by dividing the *β*-coefficient of each significant predictor by 0.25 and then by rounding off to the nearest integer value. Cox’s regression analysis was used to assess the strength of the association of covariates with mortality. First, the univariate analysis to identify potential predictors of mortality using the Kaplan–Meier survival estimates and log-rank test for each covariate. Associations that yielded a *P* value < 0.20 on univariate screen were then included in a forward regression analysis, and the strength of association between covariates and mortality was estimated by calculating the hazard ratio (HR) and 95% CIs. All survival analyses were estimated with the Kaplan–Meier test and reported as percentage ± standard error (SE) with 95% CI. All reported *P* values were two-sided; *P* value < 0.05 was considered significant.

## Results

### Study cohort

During the study period, we identified 310 (77.7%) mLEAs performed on 286 patients. This group consisted of 188 (65.7%) men and 98 (34.3%) women. Considering the entire cohort, the median age was 79 years (IQR 69–83). Demographic data, comorbidities, and risk factors are reported in Table 1. The median mFI was 4 (IQR 3–6). Indications for major amputation were as follow: CLI in 235 (75.8%), ALI in 46 (14.8%), and infection in 29 (9.4%) unrelated to PAOD. There were 163 (52.6%) primary amputations and 147 (47.4%) secondary amputations. We performed 257 (82.9%) AKA and 53 (17.1%) BKA. In 70 (22.6%) cases, a prior ipsilateral minor amputation had been performed. The intervention was performed under general anesthesia in 212 (68.4%) cases and with spinal/epidural in 98 (31.6%).

**Table 1:** Demographic data, comorbidities, and risk factors of the entire cohort (*n* = 310)

Covariate	Patients (*n* = 310)
Demographic data	
M:F (ratio)	202:108
Age (*n*, %)	
< 60 years	31 (10.0)
61–70	49 (15.8)
71–80	93 (30.0)
≥ 80 years	137 (44.2)
Comorbidity (*n*, %)	
Hypertension	263 (84.8)
Diabetes	185 (59.7)
Chronic obstructive pulmonary disease*	89 (28.7)
Coronary artery disease°	201 (64.8)
Chronic kideny disease^‡^	113 (36.5)
Hemodialysis	51 (16.5)
Congestive heart insufficiency	99 (31.9)
Atrial fibrillation	96 (31,0)
Stroke	37 (11.9)
Risk factor (*n*, %)	
Previous Vasc Surg	219 (70.6)
PAOD surgery	147 (47.4)
Previous ipsilateral minor amputation	70 (22.6)
mFI (median, IQR)^§^	4 (3–6)
BMT ongoing	194 (62.6)
Blood tests	
Hemoglobin, mean ± SD (range; g/dL)	10.2 ± 1.8 (8.9–13.7)
Leukocytes, mean ± SD (range; 10^9^/L)	15.1 ± 5.0 (3.64–41.7)
C-reactive protein, mean ± SD (range; mg/dL)	230 ± 79 (2.8–464.5)
Albumin, median (IQR, g/dL)	1.8 (0.8–2.28)

*M* male; *F* female; *n* number; *SD* standard deviation; *IQR* interquartile; *Vasc Surg* Vascular Surgery history; *PAOD* peripheral arterial occlusive disease; *mFI* modified Frailty Index; *BMT* best medical therapy

^*^Am J Respir Crit Care Med 2013;187:347–365

**°**Am J Kidney Dis 2014;63:713–735

^‡^J Vasc Surg 2016;64:e1–e21

^§^J Vasc Surg 2017;65:804–811

### Early outcomes (< 30 days)

**Fig. 2:**
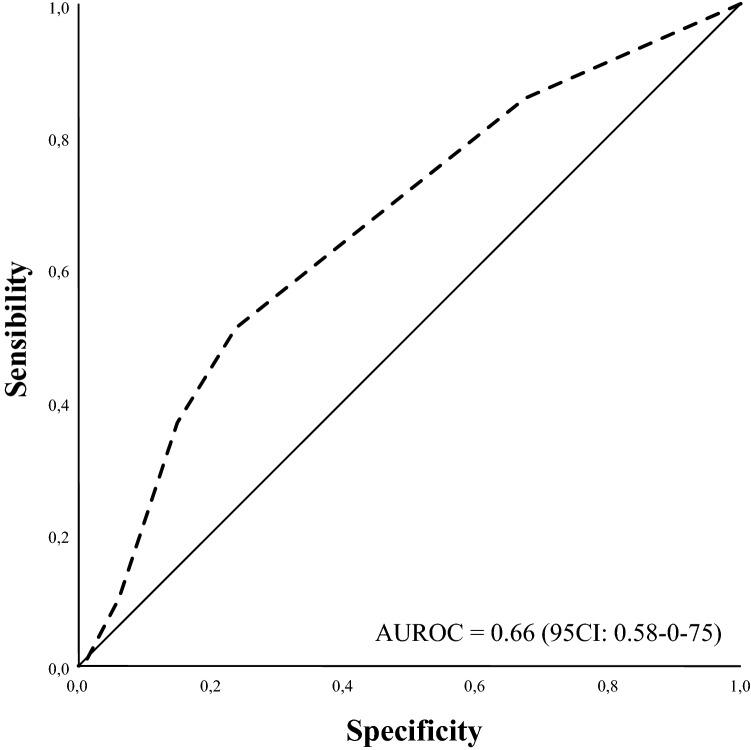
Receiver operating characteristic curve for the multivariate model evaluating the risk score for mortality at 30 days (*AUROC* area under the receiver operating characteristic curve)

There were no intraoperative deaths. Duration of the intervention was < 60 min in 158 (50.9%) patients and > 60 min in 152 (49.1%). The median length of hospitalization was 8 days (IQR 5–15 days). Complications were observed in 42 (13.5%) cases, which are described in Table 2. An intervention performed for Rutherford stage 5–6 (OR 10.3, 95% CI 2.20–47.76; *P* = 0.003) and BKA (OR 3.88, 95% CI 1.58–9.54; *P* = 0.012) was independently associated with the development of a postoperative complication. Early death occurred in 49 (15.8%) patients with the causes of death listed in Table 3. Early mortality did not differ among the different quartiles of age (15.4% vs. 14.3% vs. 15.4% vs. 19.5%, *P* = 0.826). Binary logistic regression analysis identified three predictive variables associated with 30-day mortality: age > 80 years (OR 2.24, 95% CI 1.17–4.31; *P* = 0.015), chronic obstructive pulmonary disease (OR 2.12, 95% CI 1.11–4.06; *P* = 0.023), and hemodialysis (OR 2.52, 95% CI 1.15–5.52; *P* = 0.021), listed in Table 4. The integer score assigned to each covariate was used to calculate an individual risk score for mortality at 30 days. The score ranged from 0 to 10 (median 3; IQR 0–4) owing to the sum of the three predictors (Table 5). On the basis of the assigned score, we identified two subgroups with varying mortality rates at 30 days: a lower-risk subgroup (score < 4, 10.8%) and a higher-risk subgroup (score ≥ 4: 28.7%; OR 3.2, 95% CI 1.63–6.32; *P* < 0.001 compared to the lower-risk group). The ROC analysis (AUROC 0.66, 95% CI 0.58–0.75) had reasonably good discrimination for the obtained multivariable model (Fig. 2). None of the blood tests nor operative variables were significantly associated with the development of a complication.

**Table 2:** Postoperative complication classified with the Clavien-Dindo severity grade system

Severity grading*	Complication (type)	Events (*n*, %)
Grade I/I_d_	Surgical site infection	19 (6.1)
	Wound dehiscence	7 (2.2)
Grade II	Pneumonia	2 (0.6)
	Pulmonary oedema	2 (0.6)
Grade III_b_	Wound infection	4 (1.2)
	Ab ingestis	1 (0.3)
	Wound dehiscence	1 (0.3)
Grade IV_a, b_	Septic shock	1 (0.3)
	ARDS	1 (0.3)
Grade V	Septic shock	3 (0.9)
	Cardiogenic shock	1 (0.3)

*N* number; *ARDS* acute respiratory distress syndrome

^*^Ann Surg 2004;240: 205–213

**Table 3:** Causes of early death

Cause of death	*n* = 49 (%)
Cardiovascular	
AMI/CHI/PE/GI infarction	25 (51)
Multiple organ failure	14 (28.6)
Sepsis	
Septic shock/pneumonia	4 (8.2)
Renal	
AKI/acute on CKD	3 (6.1)
Respiratory	
ARDS/acute on COPD	3 (6.1)

*AMI* acute myocardial infarction; *CHI* congestive heart insufficiency; *PE* pulmonary embolism; *GI* small bowel/colonic infarction; *AKI* de novo acute kidney injury; *CKD* chronic kidney disease; *ARDS* acute respiratory distress syndrome; *COPD* chronic obstructive pulmonary disease

**Table 4:** Univariate screen and multivariable analysis for early mortality and postoperative complications

Covariate	Early mortality					
	Univariate			Multivariate		
	OR	95% CI	*P*	OR	95% CI	*P*
Age ≥ 80	1.86	1.03–3.45	0.059	2.24	1.17–4.31	0.015
CKD	1.52	0.82–2.82	0.189			
Hemodialysis	1.85	0.89–3.85	0.139	2.52	1.15–5.52	0.021
COPD	1.92	1.02–3.60	0.057	2.12	1.11–4.06	0.023
BMT	0.63	0.34–1.16	0.149			
Vasc Surgery history	1.33	1.03–1.74	0.016			

Covariate	Postoperative complication					
	Univariate			Multivariate		
	OR	95% CI	*P*	OR	95% CI	*P*
BKA	1.33	1.06–1.68	0.002	3.88	1.58–9.54	0.003
CLI (stage 5–6)	2.26	1.20–3.01	0.015	10.23	2.20–47.76	0.03
Age > 80	2.23	1.23–4.03	0.001			
Hemodialysis	2.02	0.94–4.35	0.075			
BMT	2.44	1.12–5.30	0.025			
Duration intervention	2.99	1.47–6.09	0.002			

*OR* odd ratio; *CI* confidence interval; *CKD* chronic kidney disease; *COPD* chronic obstructive pulmonary disease; *Vasc Surgery* Vascular Surgery history; *BKA* below-the-knee amputation; *CLI* critical limb ischemia; *BMT* best medical therapy

**Table 5:** Proposed preliminary score for early mortality estimate

Covariate	*β*-coefficient	Integer score calculation	
		Yes	No
Age ≥ 80	0.81	3	0
Hemodialysis	0.92	4	0
COPD	0.75	3	0

*COPD* chronic obstructive pulmonary disease

### Late outcomes

**Fig. 3:**
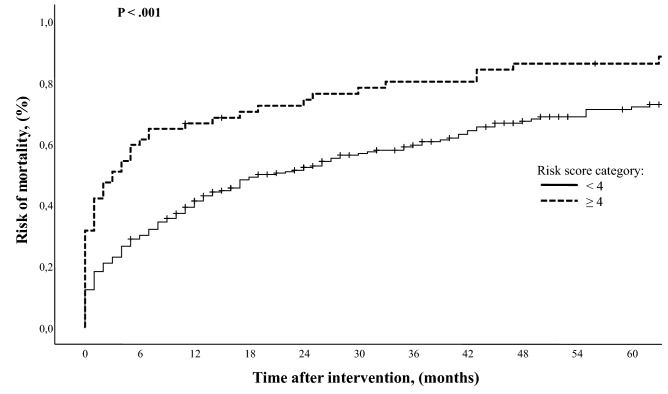
Risk analysis stratified by risk score categories

**Fig. 4:**
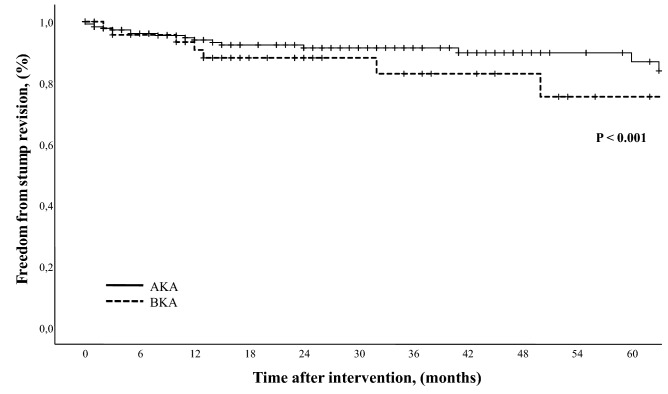
Kaplan–Meier estimates of survival stratified by level of amputation (*AKA* above-the-knee; *BKA* below-the-knee)

During the follow-up period, 175of the 261 (67%) patients died. The median FUI was 0.3 (IQR 0–1). Median survival was 19 months (IQR 7–43): estimated overall survival was 55.7% (SE 0.28; 95% CI 50.2–61.1) at 1-year, 36.6% (SE 0.29; 95% CI 31.1–42.4) at 3-year, and 25.4% (SE 0.28; 95% CI 20.3–31.2) at 5-year (Fig. 3). Long-term survival was different between the two categories of risk for early mortality. The risk of mortality in the higher-risk group was 1.8x (60.8% vs. 33.3%; log-rank *χ*^2^ = 12.9, *P* < 0.001) that of the lower-risk group (Fig. 3.). Long-term analysis through the Cox’s regression analysis identified four variables associated with mortality: need for AKA (HR 1.61, 95% CI 1.04–2.50; *P* = 0.032), age > 80 years (HR 1.69, 95% CI 1.28–2.24; *P* < 0.001), end-stage renal disease (HR 1.37, 95% CI 1.03–1.82; *P* = 0.028), and congestive heart failure (HR 1.60, 95% CI 1.22–2.11; *P* = 0.001). During the study time period, 24 (7.7%) patients underwent bilateral major amputation (AKA, *n* = 13; BKA, *n* = 11). Surgical revision of the amputation stump was required in 25 (8.1%) patients. Failure of a BKA to heal occurred in 3 (1.1%) cases at 2, 10, and 13 months after the initial amputation requiring conversion to an AKA. Freedom from amputation stump revision/failure was similar between BKA and AKA (Log-rank *χ*^2^ = 1.77, *P* = 0.183) as reported in Fig. 4. No preoperative and intraoperative variables were associated with the need for stump revision.

## Discussion

There are three findings of significance in this study: mortality after mLEA remains high and unchanged through the years, risk stratification is not adequately sensitive, and older age (e.g., ≥ 80) is the most concrete predictor of a major adverse outcome following a mLEA.

Year after year, many studies have reported consistently high mortality rates after mELAs, notwithstanding a more aggressive policy toward peripheral revascularization, better medical management, and preoperative optimization, in addition to anesthetic improvements [2, 17–19]. In our experience, early mortality remained unchanged for the past decade and is consistent with the 7.6–22.5% reported in several real-world experiences, not falling below 14% in the four quartiles of the period of study (Table 6) [3, 5–7, 17–27]. Our results are similar to Jones et al. [3], who analyzed 186,338 older patients with identified PAOD who underwent mLEA, namely the largest cohort published up to date. Though, there appears to have been a decline in the short-term, mortality rates were similar at the beginning and end of his study never falling below 12.7%. One must also consider the fact that nearly 63% of our patients were on best medical therapy at the time of mLEA, underscoring how truly frail are these patients [19].

**Table 6:** Summary of the literature including the largest experiences reporting on major lower extremity amputations and mortality analyses

Author	Type of study	Period of study (years)	Patients (*n*)	Mortality		
				30-days (%)	1 year (%)	5 years (%)
Jones et al. [3]	Medicare	2000–2008	186.388	13.5	43.8	
Easterlin et al. [5]	ACS-NSQIP	2005–2009	9.244	8.1		
Wise et al. [6]	Single center	2004–2013	295	9		
Fang et al. [7]	Single center	2010–2015	379	22.5		
Aulivola et al. [17]	Single center	1990–2001	788	8.6	30.3	65.3
Fortington et al. [18]	Multicenter	2010–2011	299	22	44	77
Gabel et al. [19]	VQI	2013–2015	2.939	5		
Stone et al. [20]	Single center	1999–2003	380	15.5		
Davenport et al. [21]	ACS-NSQIP	2005–2009	6.188	7.6		
Karam et al. [22]	VA-NSQIP	2005–2008	6.839	9.1		
Sha et al. [23]	Single center	2004–2009	454	9.2	30	40
Rosen et al. [24]	Single center	2007–2010	289	16.7	44	
Morisaki et al. [25]	Single center	2008–2015	106	7.6	36.5	63.4
Aljarrah et al. [26]	Single center	2012–2017	140		30.7	

*n* number; *VQI* vascular quality initiative; *VA-NSQIP* Veterans Administration National Surgical Quality Improvement Program; *ACS-NSQIP* American College of Surgeons National Surgical Quality Improvement Program

Despite the large number of mLEAs performed every year, risk stratification in this clinical context is still meager [5, 7]. Since no single clinical or physiologic parameter has been able to reliably predict a poor outcome after mLEA, the use of a risk-prediction score may be a more accurate method to optimize the risk stratification [27]. Taking advantage of the large number of patients contained in the American College of Surgeons National Surgical Quality Improvement Program, Easterlin et al. [5] aimed to create a risk index to predict 30-day mortality after mLEAs for PAOD. Their scoring system included eleven covariates and showed to have similar discriminatory power to several renowned risk scores used to predict surgical outcomes. The risk score developed from our cohort relies on fewer covariates, thus simplifying the process, but decreasing the accuracy shown by our model. However, our findings are worthy of several observations and conclusions. First, it constitutes our institutional audit, which is an important method of professional quality improvement based on examination of outcomes and correction of substandard practice [28]. Secondly, the covariates identified by our model have been already confirmed to be associated with mortality after mLEAs in several experiences, thus known and reliable predictors [1, 3, 5, 18, 29–31]. Third, the model allowed us to generate two markedly different categories of risk for early mortality, a distinction that was also associated with a significant difference in long-term survival in favor of the lower-risk group [5]. This could mean that lower-risk patients, those who are least likely to die, are the ones most likely to survive longer. In light of the fact that overall early mortality has been unchanged over the years, and the fact that patients within the lower-risk category are those who benefit from longer survival, we may have to reverse how to interpret the significance of our score model. Although our results must find future confirmation, patients who are more likely to survive the past 30-days might benefit from additional improvement of the intensity of perioperative care, which could ultimately further improve survival rates. Better perioperative care could be centered on specific risk factors, optimization of blood tests, medical therapy enhancement, and also taking advantage of delaying a non-urgent intervention [3, 6, 32].

The literature is abundant with studies confirming that older age is an important predictor for adverse outcomes [1, 3, 5, 18]. Therefore, it is not surprising that older age, specifically ≥ 80 years, has been shown to be the most important predictor of mortality following mLEAs [33]. In particular, it is interesting to note that in the index score built by Easterlin et al. [5],this same age distinction was their most powerful covariate. Despite all commendable attempts to refine risk stratification, we are undoubtedly in need of a more accurate risk prediction system. Nevertheless, non-operative management in high-risk patients should still be avoided if possible [6]. We hope our findings may lead to further initiatives on this aspect.

Amputation level remains critical to outcomes. In this study, an AKA was more frequently associated with mortality compared to a BKA, a result similar to several other investigators [3, 17, 19, 24]. Indeed, the difference between the two groups seems to be mainly determined by the risk profile of our patients. However, while prior investigators have reported that an increased perioperative mortality rate in AKA patients was associated with the presence of advanced ischemia, the most determining factor for AKA in our experience was older age [5, 19]. On one hand, it further underlines the impact of age on mLEA outcomes as BKA patients had a higher incidence of diabetes, chronic kidney disease, or hemodialysis [17, 26, 30, 31]. The association of these comorbidities with BKA is the main rationale as to why a BKA was significantly associated with postoperative complications, and more frequently required proximal revision surgery because of stump failure [17].

Considering their overall frailty status, patients needing mLEA for PAOD have been shown to be at high-risk for major adverse events. In our experience, most of the interventions have been performed under general anesthesia which can be considered a potential risk factor in these patients [34]. In our experience the type of anesthesia did not impact negatively on both major outcomes. While older studies reported a potential benefit of regional anesthesia in comparison with general anesthesia, our result data finds support in several recent experiences which reported the mode of anesthesia, did not have a significant effect on perioperative outcomes after mLEA [5, 19, 22, 29, 34]. Although it is difficult to give an unquestionable explanation, the combination of advancement in perioperative care and anesthesia management along with multidisciplinary evaluation might have been beneficial on tempering worse outcome with a specific anesthesia regimen [34].

### Limitations

The present study has several limitations. It is retrospective in nature, the sample size is small and prospective evaluation of the risk score is needed to clarify the potential utility in the decision process. Nonetheless, although large databases have significant value through increased power and sample size, a single institutional analysis may offer granular detail that may not be available in the larger study. All operations were managed by members of our service only and not by different divisions or departments. Although all these features may not allow for the generalizability of our findings, our data compare well with the available literature owing to the consistency of follow-up data validated by official health documents and will allow us to further refine our processes and perform continuous quality improvement.

## Conclusions

In our experience, mLEAs continue to be associated with a disturbingly high mortality rate that remains greater 14% over the years. Our predictive score discriminated two categories of patients with significantly different risks of early mortality and long-term survival. In particular, the group with a score > 4 is characterized by significantly higher early as well as long-term mortality: in these patients, major amputation likely represents a marker of advanced illness that significantly limit survival independently of the possibility to perform this intervention. Therefore, prospective validation will help to refine our risk stratification and treatment policy for these patients who would also potentially represent a logical population to engage in a proactive discussion of end-of-life.

## Data Availability

Fully available; surgeons are responsible for data capture, insertion, and auditing.

## Code availability

Microsoft Excel (Microsoft Corp—Redmond; Wash—USA).
